# Hepatitis B Virus Infection Among Tribal Populations in India: A Systematic Review and Meta-Analysis

**DOI:** 10.3389/phrs.2025.1607620

**Published:** 2026-03-02

**Authors:** Abhinav Sinha, Gayatree Nanda, Rounik Talukdar, K. Divyasree Bhat, Banamber Sahoo, Chandrakant Lahariya, Sanghamitra Pati, Prakash Kumar Sahoo

**Affiliations:** 1 ICMR-Regional Medical Research Center, Bhubaneswar, India; 2 ICMR-National Institute of Cholera and Enteric Diseases, Kolkata, West Bengal, India; 3 Foundation for People-Centric Health Systems, New Delhi, India

**Keywords:** Hepatitis B, systematic review, meta-analysis, India, tribal

## Abstract

**Objectives:**

The introduction of the Hepatitis B virus (HBV) vaccine has significantly reduced the disease’s burden. Tribes comprise approximately 8.6% of the Indian population, making it pertinent to investigate the epidemiology of HBV among these individuals. We synthesized the prevalence of HBV among tribes in India.

**Methods:**

We searched the Medline (via the PubMed search engine), Embase, and CINAHL databases, in addition to the first 10 pages of Google Scholar. We included original observational studies that screened tribal populations for HBV infection, reported the prevalence of HBsAg (our main preference), and/or other markers. The risk of bias was assessed using the Appraisal Tool for Cross-Sectional Studies. The pooled prevalence was presented after conducting a meta-analysis (PROSPERO registration ID: CRD42022334938).

**Results:**

A total of 24 studies were selected for this study. The pooled prevalence of HBV (as measured by the proportion of individuals testing positive for hepatitis B surface antigen) was estimated to be 9.99% (95% confidence interval (CI) 6.07–14.75, I^2^ = 98.7%, p < 0.01). The highest HBV prevalence was noted in the northern zone of the country (19.60%, 95% CI 15.09–24.54, I^2^ = 84.4%, p < 0.01), followed by the northeastern zone (13.43%, 95% CI 6.09–23.08, I^2^ = 98.4%, p < 0.01), and the southern zone (10.44%, 95% CI 4.75–18.01, I^2^ = 98.9%, p < 0.01).

**Conclusion:**

A considerable prevalence of HBV was observed in tribal communities in India, a fact that cannot be overlooked. This information may be useful for planning HBV vaccination strategies among tribes in India.

## Introduction

Hepatitis B virus (HBV) infection is a common infection that affects the liver and is caused by the HBV, a type 1 DNA virus of the Hepadnaviridae family [1]. Globally, Hepatitis B remains a public health concern and a leading cause of liver cirrhosis, hepatocellular carcinoma, and chronic liver disease, resulting in an estimated 820,000 deaths per year [1]. HBV is transmitted through contaminated blood, semen, and other bodily fluids [1]. Additionally, it can be transmitted vertically from mother to child during delivery, through HBV-contaminated blood products, such as those used while performing medical procedures, and through needles shared among drug users [1]. Individuals who have multiple sexual partners are at a higher risk of contracting the infection [1]. According to the 2019 data from the World Health Organization (WHO), approximately 296 million people had chronic HBV infection, with nearly 1.5 million new infections occurring annually [1]. A meta-analysis published in 2007 observed the prevalence of HBV to be approximately 1.46% and categorized India as a low endemicity zone with approximately 17 million chronic carriers [2].

According to the 2011 census, there are approximately 104.3 million individuals living in tribal communities in India, which is approximately 8.6% of the total Indian population [3]. A 2009 meta-analysis conducted in India using population weights showed a prevalence of 11.85% among tribal communities [4].

 According to Narain (2019), “Tribal communities in India have poor health indicators, greater burden of morbidity and mortality and very limited access to healthcare services. There is also a near complete absence of data on health situation of different tribal communities.” [5].

Many tribal communities reside in remote and difficult-to-reach areas with inadequate transportation and poor health infrastructure, resulting in low coverage of routine immunization and poor uptake of preventive services [6]. Scarcity of health workers, irregular outreach activities, and frequent stock-outs of vaccines and supplies further hinder the timely delivery of the HBV vaccine, especially the birth dose, which is essential to preventing vertical transmission [7]. Multiple studies have documented the poor health status of tribal populations across various regions of India [8–10]. Additionally, traditional cultural practices such as tattooing, body piercing, scarification, and the use of shared or unsterilized instruments for minor surgical procedures or traditional healing rituals can facilitate the transmission of HBV through blood exposure [11]. Furthermore, these practices are often carried out outside of formal healthcare settings and are deeply rooted in cultural beliefs, so they may not be perceived as health risks by the community [11]. The lack of awareness about HBV transmission, combined with low health literacy and limited engagement with public health campaigns, compounds the vulnerability of these groups [12]. Together, these structural and sociocultural factors create a setting in which HBV transmission can persist despite the availability of preventive measures, such as vaccination. However, the present meta-analysis data are outdated, as evidence suggests a reduction in the burden of HBV infection among tribal populations due to the Hepatitis B vaccination program introduced in India in 2010–2011 [13]. A recent study conducted among 2,737 individuals from 35 tribal communities and five particularly vulnerable tribes in Odisha observed an HBsAg positivity rate ranging from 1.79% to 2.94% [14]. Therefore, new data is crucial for guiding public health strategies, particularly for the implementation of the post-vaccination program.

The WHO adopted the Global Health Sector Strategy on viral hepatitis (2016–2021) during the World Health Assembly in 2016, which aimed to eliminate viral hepatitis as a public health threat by 2030. Therefore, there is an urgent need to synthesize recent data on the burden of HB among tribal communities, which will help with the proper management and prevention of HBV. This evidence will also be useful in guiding the future course of the vaccination program and strategies by allocating resources to vulnerable groups. We systematically reviewed and pooled the prevalence of HBV infection among India’s tribal populations and mapped the evolution of biotechnology-related serological tests for estimating hepatitis B infection.

## Methods

### Standards and Protocols

The present review was registered with the International Prospective Register of Systematic Reviews (PROSPERO ID: CRD42022334938) [15]. We followed the Preferred Reporting Items for Systematic Review and Meta-Analysis (PRISMA) guidelines to perform and present this review (Supplementary Table 1) [16].

### Eligibility of Studies

Observational studies that reported the data on community-based prevalence of Hepatitis B among tribal populations in India, including short reports, were included. Qualitative studies, reviews of any kind, meta-analyses, conference abstracts, and editorials were excluded.

### Types of Participants/Population

Studies conducted in India that documented the prevalence of HBV infection among healthy subjects belonging to tribal communities and reported the prevalence of HBsAg (our main preference) and/or other markers were included in this study. Individuals with sexually transmitted infections were excluded, as we wanted to estimate the prevalence of HBV in healthy individuals.

### Information Sources

Three electronic databases, Embase, Medline via the PubMed search engine, and CINAHL, were systematically searched. Additionally, to make the search more comprehensive, the first 10 pages of Google Scholar were searched, and the references of the included studies were also searched manually.

### Search Strategy

We used three major concepts: “Hepatitis B″, ‘Tribal’ and ‘Prevalence’. PubMed was used to create an exhaustive search syntax, which was later adopted for the other two databases. MeSH terms were employed for PubMed, whereas Emtree terms were used for Embase. The MeSH terms utilized were “hepatitis B”[MeSH] and “Indigenous Peoples”[MeSH] for the respective major concepts. The relevant keywords were identified from the literature to make the search more comprehensive. All three concepts were joined using the Boolean operator “AND.” We did not apply any other restrictions, such as language, but we considered studies published until April 2025. The detailed search strategy for each of the databases is provided in Supplementary Table 2.

### Study Selection and Data Management

Two reviewers (AS and GN) independently conducted the primary screening based on the titles and abstracts of the articles and categorized the studies as included, excluded, or unsure. Studies deemed irrelevant by both reviewers were excluded. Next, the full texts of the included articles were thoroughly screened by two reviewers (AS and GN) on the basis of pre-established inclusion and exclusion criteria. Any differences between the two reviewers were resolved by the entire team through consensus. Data, including the first author’s name, publication year, sociodemographic characteristics (such as age and sex), study location, name of tribes, study setting, study period, sampling method, testing method, markers to determine the prevalence of Hepatitis B (e.g., HBsAg, HBV DNA, Anti-HBc, Anti-HBs, and HBeAg), and the reported risk factors were extracted from included studies by two independent reviewers (AS and GN). Before entering the data into a pre-formatted data extraction sheet, two reviewers (PKS and SP) further verified the data.

### Risk of Bias (Quality) Assessment

The quality of the studies was independently assessed by two reviewers (AS and PKS) using the evaluation tool for cross-sectional studies (AXIS). This tool consists of 20 questions from five key domains, such as introduction, methods, results, discussion, and others [17]. The key domains address questions related to study design, sample size justification, target population, sampling frame, sample selection, and methods, among others. Each question was marked as “yes = 1” or “no/do not know = 0,” and studies obtaining 0%–50% were reported to have “high risk of bias,” 51%–80% as “medium risk of bias,” and 81%–100% as “low risk of bias.” Any disagreement about the risk of bias was resolved in consultation with PKS. The AXIS tool’s detailed questions are presented in Supplementary Table 3.

### Data Synthesis

A qualitative and quantitative summary of the data was produced. The qualitative summary included the characteristics of the included studies along with their basic description. R Studio (version 4.1.3, 2022; The R Foundation for Statistical Computing, Vienna, Austria) was used to perform the meta-analysis. The “metaprop” package (Rdocumentation.org) was used to perform the meta-analysis of single proportions. Proportional variances between studies were stabilized using the Freeman-Tukey double arcsine transformation. The standard error (SE) for the proportions was computed based on the extracted data. The formula used for the calculation was: “SE = √ (P × (1–P)/n),” where P represents the proportion and n is the population size. The meta-analysis was conducted using a random-effects inverse-variance model, which included calculating a weighted average using standard errors (SEs) and supplying the data frame for the “metaprop” package in R Studio. We presented the pooled estimates as percentages with 95% confidence intervals (CIs), which were represented by a forest plot. I^2^ statistics were used (I^2^ statistics: 25% - mild, 25%–75% - moderate, and >75% - high) to measure heterogeneity across studies [18]. Additionally, a subgroup analysis was performed based on the administrative zones in India to account for geographic variability. To further address the issues of high heterogeneity between studies, a prediction interval was provided with each pooled estimate. A sensitivity analysis was performed using the Leave-one-out method, which enabled us to predict the variation in the pooled estimates and the stability of the model [19]. We constructed a funnel plot to analyze the publication bias, examining it through an Egger’s test, where a p-value of ≤0.05 was deemed to be statistically significant.

### Ethical Considerations

This review was conducted based on the published literature, thus eliminating privacy concerns.

## Results

### Search Results

**FIGURE 1:**
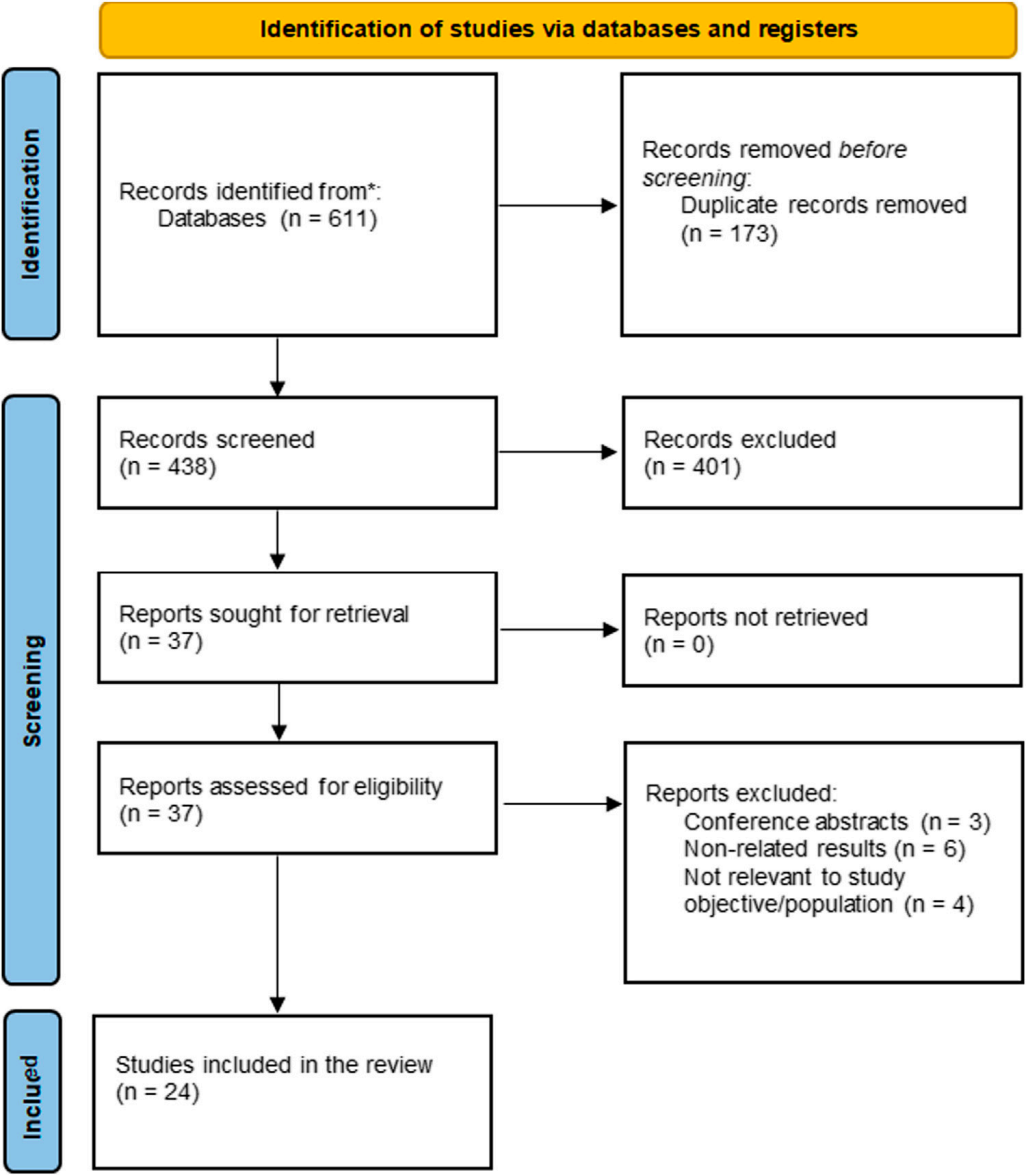
Flowchart depicting the selection process for the included studies.

A total of 611 articles were identified by searching the three databases indicated above and by conducting manual searches (cross-referencing). We removed 173 duplicate studies (Figure 1). Furthermore, the full texts of 37 articles were reviewed after primary screening based on titles and abstracts. A total of 24 studies finally fulfilled the inclusion criteria and were selected for the study [20–44].

### General Characteristics of the Included Studies

Of the 24 included studies, seven were conducted in the Andaman and Nicobar Islands, making it the region with the highest number of studies on HBV prevalence among tribal populations. [34–37]^,^ The age of the participants ranged between 0.04 years of age and 90 years of age. Reporting of participant sex was limited to 18 studies; the other studies did not present corresponding data (Table 1) [14, 20–42].

**TABLE 1:** Characteristics of the included studies (India, 1995–2025).

Author name, year	Study site	Sample size	Male	Sampling method (Random/Non-Random)	Study setting (community/Facility)	Age range (years)	Study period
Anvikar et al. [20]	Madhya Pradesh	526	274	Random	Community	15–49	2004 to 2005
Barall et al. [21]	Himachal Pradesh	1,110	424	Random	Community	0–90	2015 to 2016
Bhattacharya et al. [22]	Andaman and Nicobar Islands	612	227	Non-random	Community	Mean Age 41.2 ± 1.1	2010 to 2011
Bhattacharya et al. [28]	Andaman and Nicobar Islands	726	NA	Non-random	Community	NA	NA
Bhattacharya et al. [30]	Andaman Nicobar	726	313	Random	Community	NA	2010 to 2012
Bhattacharya et al. [41]	Odisha	4,006	1786	Random	Community	1–93	2022–2023
Bhattacharya et al. [14]	Odisha	2,737	1,176	Random	Community	>6	NA
Bhaumik et al. [37]	Tripura	3,475^a^	1,582	Random	Community	10–90	2011 to 2013
Biswas et al. [23]	Arunachal Pradesh	438	198	Random	Community	2–57	NA
Borkakoty et al. [24]	Arunachal Pradesh	438	NA	Random	Community	2–56	2005
Dinesh et al. [25]	Tamil Nadu	372	118	Random	Community	≥18	NA
Dwibedi et al. [26]	Odisha	1765	NA	Random	Community	1–45	2006 to 2010
Ghosh et al. [40]	Jharkhand	737	NA	Random	Community	<10	NA
Gnanasekaran et al. [27]	Tamil Nadu	72	34	Non-random	Community	≥18	2008
Habeeb et al. [29]	Andhra Pradesh	890	NA	NA	Community	NA	NA
Haldipur et al. [31]	Arunachal Pradesh	1,077	1,077	NA	Community	19–49	1963
Murhekar et al. [32]	Andaman and Nicobar Islands	1,266	535	Random	Community	5–80	1998 to 1999
Murhekar et al. [33]	Andaman and Nicobar Islands	887	437	Random	Community	<45	NA
Murhekar et al. [34]	Andaman and Nicobar Islands	1,574	817	Non-random (Census)	School	5–14	NA
Murhekar et al. [35]	Andaman and Nicobar Islands	64	40	NA	Community	5–14	NA
Reddy et al. [38]	Madhya Pradesh	91	40	Random	Community	NA	NA
Sharma et al. [37]	Himachal Pradesh	1,327	618	Random	Community	All age groups	2017
Shyamala et al. [39]	Kerala	240	86	NA	Community	17–85	2014 to 2015
Sahoo et al. [42]	Odisha	263	141	Random	Community	7 months old to 5 years old (Children)	2020–21

^a^ Excludes Bengali Hindus and Muslims and includes only tribal participants; NA, Not Available.

The HBsAg status was reported in all studies.1 [14, 20–42] Along with HBsAg, the HBV DNA status, which was analyzed using polymerase chain reaction (PCR), was reported by seven studies. The prevalence of HBeAg was reported in seven studies, while anti-HBs and anti-HBc were detected in 15 and 14 studies, respectively, with 11 studies containing data from both (Table 2). Regarding the testing method, the enzyme-linked immunosorbent assay (ELISA) was performed in 22 studies, while one of the studies used the Enzyme Linked Immuno-Filtration Assay (ELIFA) and one used an enzyme immunoassay (EIA) (Supplementary Table 4).

**TABLE 2:** Prevalence of Hepatitis B virus infection reported in the included studies (India, 1995–2025).

Author name and year	Tribes	Total samples	HBsAg n (%)	HBV DNA	Anti- HBs n (%)	Anti-HBc n (%)	HBeAg n (%)
Anvikar et al. [20]	NA	526	15 (2.9)	NA	NA	NA	NA
Barall et al. [21]	NA	1,093	241 (22.05)	NA	NA	NA	NA
Bhattacharya et al. [22]	NA	612	54 (8.82)	62 (10.1)	12/62 (19.4)	18/62 (29)	NA
Bhattacharya et al. [28]	NA	726	35/82 (42.7)	98 (13.5)	NA	NA	11/82 (13.41)
Bhattacharya et al. [30]	Vaccinated Nicobarese, Non- Non-Vaccinated Nicobarese	211 515	5 (2.4), 49 (9.5)	NA	180 (85.3) 276 (53.6)	128 (60.6), 229 (57.1)	NA
Bhaumik et al. [36]	Reang	455	35 (7.69)	NA	NA	NA	NA
	Chakma	184	21 (11.41)				
	Debbarma	531	13 (2.45)				
	Halam	522	22 (4.21)				
	Jamatia	526	30 (5.7)				
	Lusai	111	3 (2.7)				
	Murasing	272	14 (5.15)				
	Noatia	115	7 (6.09)				
	Tripuri	283	14 (4.95)				
	Other tribes	476	4 (0.84)				
Biswas et al. [23]	Idu Mishmi	438	93 (21.2)	NA	96/200 (48)	193/209 (92.3)	34/93 (36.6)
Borkakoty et al. [24]	Idu Mishmi	438	93 (21.23)	36/93 (38.7)	NA	NA	NA
Dinesh et al. [25]	Irula	372	19 (5.1)	NA	NA	NA	NA
Dwibedi et al. [26]	Lodha	242	2 (0.8)	NA	4 (1.6)	11 (4.5)	NA
	Juanga	460	8 (1.7)	NA	44 (9.5)	74 (16.1)	
	Khadia	450	4 (0.9)	NA	28 (6.2)	42 (9.4)	
	Mankidia	401	15 (3.8)	NA	37 (9.3)	105 (26.3)	
	Saora	212	2 (0.9)	NA	2 (0.9)	3 (1.4)	
Ghosh et al. [40]	Paharias	737	15 (2)	11/15 (73.3)	NA	NA	4/15 (26.6)
Gnanasekaran et al. [27]	Irula	72	8 (11.11)	NA	NA	NA	NA
Habeeb et al. [29]	Lambada	890	46 (5.16)	9/46 (19.5)	NA	NA	9/46 (19.5)
Haldipur et al. [31]	NA	1,077	159 (15)	29/150 (19)	NA	458 (44)	NA
Murhekar et al. [32]	Nicobarese	1,144	267 (23.3)	NA	210/877 (23.9)	NA	NA
	Shompens	37	14 (37.8),	NA	1/23 (4.3)		
	Onges	58	18 (31),	NA	10/40 (25)		
	Andamanese	27	1 (3.7)	NA	4/26 (15.4)		
Murhekar et al. [33]	Tamalo	887	197 (22.2)	NA	171/651 (26.3)	259/480 (54)	35/190 (18.4)
Murhekar et al. [35]	Nicobarese	1,574	354 (22.5)	NA	NA	NA	NA
Murhekar et al. [34]	Jarawas	64	42 (65.6)	NA	NA	44 (68.8)	NA
Reddy et al. [31]	Baiga	91	4 (4.4)	NA	NA	30 (33)	NA
Sharma et al. [37]	Lahaul	616	19/313 (6.1)	NA	NA	NA	NA
	Spiti	711	122 (17.2)	NA	NA	NA	NA
Shyamala et al. [39]	Kurichiya	240	Null	NA	NA	NA	NA
Bhattacharya et al. [41]	Bondo Poraja	530	20 (3.75)	NA**	NA**	NA**	NA**
	Chuktia Bhunjia	576	6 (1.06)				
	Didayi	261	3 (1.06)				
	Dongria Khond	463	40 (8.73)				
	Juang	332	6 (4.98)				
	Kharia	99	3 (3.13)				
	Kutia Khond	183	3 (1.78)				
	Lanjia Saora	596	22 (3.83)				
	Lodha	384	14 (3.74)				
	Mankidia	122	2 (1.76)				
	Paudi Bhuyan	458	19 (4.30)				
Bhattacharya et al. [14]	Bhatara	22	Null	NA**	NA**	NA**	NA**
	Bhatra	340	3 (0.88)				
	Bhuiya	36	Null				
	Bhuyan	64	2 (3.13)				
	Gond	100	6 (6.00)				
	Gondo	50	2 (4.00)				
	Juang	64	Null				
	Khond	333	12 (3.60)				
	Kisan	152	Null				
	Kol	47	2 (4.26)				
	Kolha	47	0				
	Kond	176	1 (0.57)				
	Kora	21	0				
	Munda	128	1 (0.78)				
	Oraon	65	0				
	Rajuar	16	1 (6.25)				
	Santal	34	0				
	Saora	25	0				
	Savar	297	7 (2.36)				
	Sounti_Bhumia	29	0				
	Other tribes (Bhathudi, Bhumij, Binjhal, Dadua, Didayi, Gadaba, Ghara, Kawar, Kharia, Kharwar, Korua, Koya, Madia, Mundari, Paroja, among others)	366	2 (0.55)				
	Dongria_Kondh	1	1 (100)				
	Kutia_Khond	141	20 (14.18)				
	Lanjia_Saora	1	0				
	Paudi_bhuyan	165	10 (6.06)				
	Saora	17	0				
Sahoo et al. [42]	Not specifically mentioned	263	3 (1.14)	N/A	N/A	N/A	N/A

*NA, Not available/Not reported; Data from samples with fewer tests are presented as n/total samples tested (%); NA**.

### Prevalence of Hepatitis B

The HBV prevalence among the Indian tribal population was found to range from 0% in the Kurichiya tribes of Kerala to 65% in the Jarawa tribes of the Andaman and Nicobar islands. A very high level of HBsAg positivity was also reported among the tribes of the Andaman and Nicobar islands (ranging from 23.3% in the Nicobarese tribe to 37.8% in the Shompen tribe). The point prevalence of HBsAg was found to be 21.2% among the Idu Mishmi tribes of Arunachal Pradesh. However, the most recent HBV status report found the prevalence of HBV to be 3.6% (HBsAg positivity) in the tribes of Arunachal Pradesh. Among the Tripura tribal populations, the highest and lowest prevalence rates were observed among the Chakma (11.41%) and Lusai (2.7%) tribes, respectively. Low levels of HBsAg prevalence were observed in the primitive tribes of Odisha, ranging from 0.8% in the Lodha tribe to 3.8% in the Mankidia tribe. Among the Spiti tribes of Himachal Pradesh, HBsAg positivity was reported at 17.2%–22.05%, whereas it was observed to be 2% among the Paharias of Jharkhand and at 2.9%–4.4% in Madhya Pradesh, where the specific tribal groups were not reported. The lowest prevalence levels were observed in the Didayi and Chuktia Bhunjia tribes (1.06% each) and among the Mankidia (1.76%) and Kharia (3.13%) tribes. The Kutia Khond tribe showed the highest HBsAg positivity at 14.18%, followed by the Paudi Bhuyan tribe (6.06%) and the Gond tribe (6.00%). Other tribes, such as the Khond (3.60%), Bhuyan (3.13%), and Kol (4.26%), were found to have moderate HBsAg positivity levels. A more recent assessment by Sahoo et al. among an unspecified tribal population in Odisha showed a relatively low HBsAg positivity rate of 1.14% (Table 2). The presence of HBeAg in the serum ranged from 7.14% in the Attapady tribal population of Kerala to 36.6% in the Idu Mishmi tribe of Arunachal Pradesh. In total, 85.5% of the vaccinated Nicobarese individuals were anti-Hbs positive, indicating protection against HBV. A total of 92.3% of the Idu Mishmi tribe were anti-HBc positive, indicating a past or current Hepatitis B infection. Cases of Occult Hepatitis B infection (OBI) were also found in 11.1% of the Nicobarese tribe tested for HBV DNA (Table 2).

**FIGURE 2:**
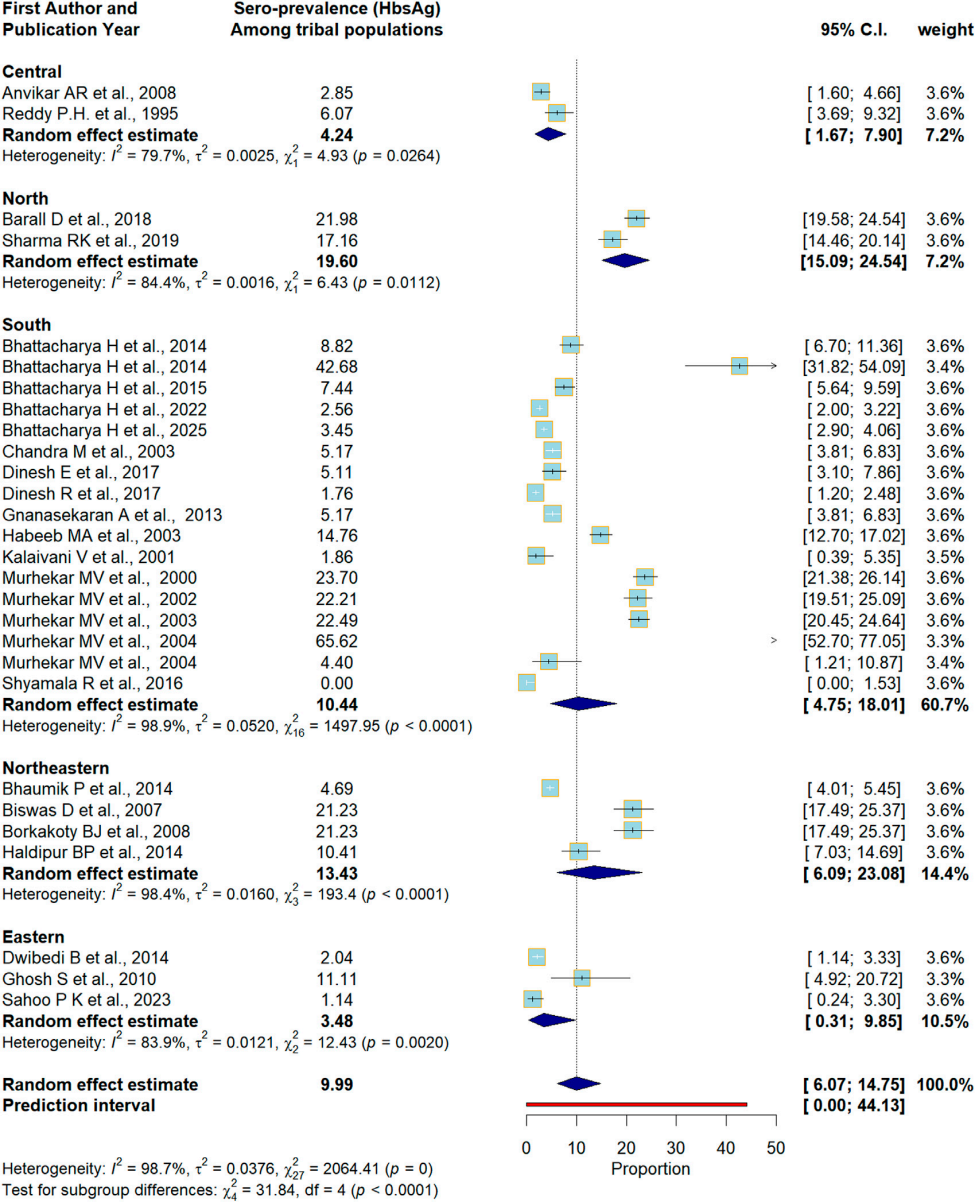
Forest Plot showing the pooled prevalence of Hepatitis B surface Antigen positive cases; Zone-wise subgroup analysis (India, 1995–2025). Note: Wide prediction interval. Although the test of subgroup difference is significant (p < 0.01), residual heterogeneity exists in some of the subgroups. In the central and eastern zones, heterogeneity drops significantly within subgroups, which explains the effect of geographic location on the estimate to some extent.

The HBV DNA was reported on an individual basis, not a tribal basis. The nationwide pooled prevalence of HBV (as measured by hepatitis B surface antigen positivity) was estimated to be 9.99% (95% confidence interval (CI) 6.07–14.75, I^2^ = 98.7%, p < 0.01) with a prediction interval ranging from 0.00 to 44.13 (Figure 2). To account for the high heterogeneity, subgroup analysis was performed based on disaggregated estimates of HBV prevalence in different administrative zones in India. The highest HBV prevalence was noted in the northern zone of the country (19.60%, 95% CI 15.09–24.54, I^2^ = 84.4%, p < 0.01), followed by the northeastern zone (13.43%, 95% CI 6.09–23.08, I^2^ = 98.4%, p < 0.01) and the southern zone (10.44%, 95% CI 4.75–18.01, I^2^ = 98.9%, p < 0.01). Residual heterogeneity was found to persist within subgroups after disaggregated analysis, although the test of subgroup difference was statistically significant (p < 0.01).

**FIGURE 3:**
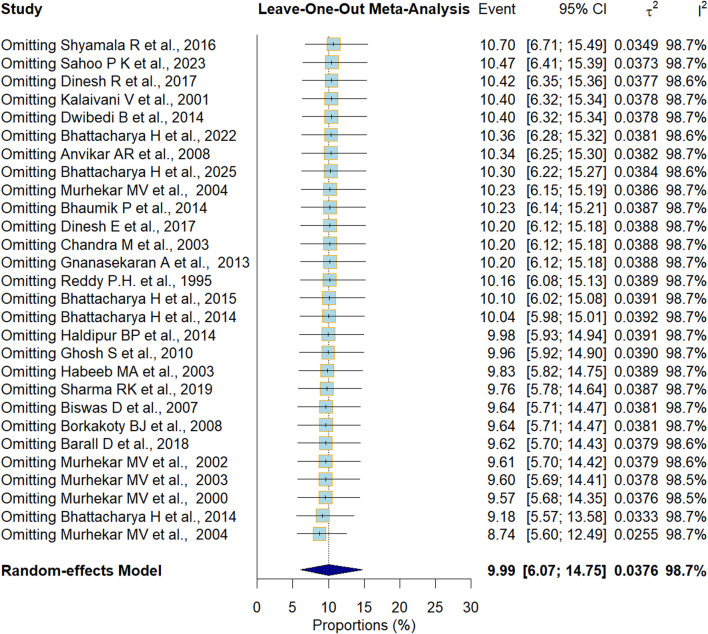
Sensitivity Analysis (India, 1995–2025). No single study had any untoward effect on the pooled estimate for Hepatitis B surface Antigen.

The non-significant Egger’s p-value (0.56) and visual inspection of the funnel plot indicated that publication bias is unlikely (Supplementary Figure 1). To assess the robustness of our HBsAG national prevalence estimate, a leave-one-out analysis was performed. The pooled estimates, when each study was omitted one at a time, ranged from 10.70% to 8.74%, while the overall study estimate was 9.99% (Figure 3).

### Risk of Bias Assessment

We observed that 21 studies had a moderate risk of bias [14, 20–23, 25, 26, 28, 30–42], while three studies [24, 27, 29] had a high risk of bias (Supplementary Table 5).

## Discussion

### Key Findings

We observed a significant pooled prevalence of Hepatitis B among tribal populations. The majority of the included studies used ELISA to test for Hepatitis B. The majority of the studies measured HBsAg to report the burden of Hepatitis B, while ELISA was the most commonly conducted serological test.

### Comparison With the Existing Literature

We observed wide diversity in HBV prevalence among different tribes, ranging from 0% (Kurichiya tribes of Kerala) to 65% (Jarawa tribes of the Andaman and Nicobar islands). However, a recent systematic review reported that the prevalence of HBV infection in the general population ranged from 0.87% to 21.4%, which is lower than the findings of the present study [44]. This further strengthens the idea that tribal communities are a vulnerable group and should be the focus of HBV-related interventions. We reported the pooled prevalence of HBV infection to be approximately 11.09%, which is lower than the 15.9% HBV prevalence reported in a meta-analysis conducted in 2007 among tribal populations in India [4, 6]. Additionally, our recent study conducted among under-five children from tribal communities also observed the prevalence of HBV to be low (1.14%), which further supports the notion that the introduction of a vaccine against HBV in routine immunization programs has resulted in a steady decrease in HBV burden [45]. Nonetheless, tribal populations remain vulnerable to this infection, which highlights the need for continued vaccination strategies along with efforts to educate these communities about the infection and its transmission routes so that Hepatitis B can be prevented.

The present review found ELISA to be the most commonly used serological test for the detection of HBV, which is consistent with the findings of a systematic review published in 2015 that also reported ELISA to be the most common HBV diagnostic method [46]. A probable reason for this could be the popularity and the cost-effectiveness of ELISA. Nonetheless, other novel and highly sensitive techniques, such as flow cytometric quantification and covalently closed circular DNA (cccDNA), may be used for diagnosing the etiology of chronic hepatitis and predicting the recurrence of disease, respectively [47].

There are approximately 550 different tribes residing in different regions and spread throughout India [47]. Their subsistence largely depends on farming. This population has distinct sociocultural practices such as tattooing, pricking, and piercing of body parts, which are important risk factors for HBV infection. These sociocultural practices, along with their living conditions, sexual promiscuity, higher rates of poverty, and limited literacy, make this group vulnerable to HBV [1]. This highlights the need to raise awareness among the general public about the possible routes of HBV transmission, which include exposure through the mucosal membrane, body fluids, and perinatal transmission. Moreover, HBV infection may lead to chronic liver diseases such as cirrhosis and hepatocellular carcinoma. Furthermore, individuals who have ever been infected with HBV may be at risk for viral reactivation, which occurs when host immunity interplays with HBV replication, necessitating complete treatment of infected individuals. However, limited data did not allow us to assess the proportion of subjects in need of treatment, which should be assessed in future studies. Nonetheless, a systematic review reported that there may be 12%–25% of patients requiring treatment in various settings, i.e., community and clinical.

### Implications for Policy and Practice

To eliminate viral hepatitis as a public health threat by 2030, one of the critical actions is to identify and prioritize high-burden and vulnerable populations for targeted interventions. Given the considerable and disproportionate burden of Hepatitis B observed among tribal populations in this review, this group clearly falls into that high-priority category. This can directly inform national and subnational immunization strategies and can guide the equitable allocation of resources under the broader umbrella of India’s commitment to the GHSS targets. The integration of tribal-specific vaccination plans, supported by decentralized health governance and resource deployment, is essential. Moreover, the identification of traditional practices as risk factors underscores the need for culturally sensitive programming. Additionally, Information, Education, and Communication (IEC), along with Behavior Change Communication (BCC) strategies, are required. Culturally appropriate and linguistically tailored messages should be provided to tribal communities so that they can understand and adopt safe practices, especially with regard to the use of injections. IEC materials may include ‘fotonovelas’ or books containing photos showing how HBV infection spreads. Nonetheless, vaccination remains a key to controlling the infection, and it should reach all segments of society. Although India has high vaccination coverage, these populations should still remain the focus.

### Strengths and Limitations

We conducted a thorough literature search in three databases. Additionally, we followed a prospectively registered protocol to conduct this review. Two independent reviewers assessed screening, data extraction, and risk of bias, which gave additional methodological rigor to the review. However, the paucity of data on vaccinated and non-vaccinated individuals did not allow us to synthesize and compare data based on vaccination status, which could have been useful to inform new programs and policies.

## Conclusion

A considerable prevalence of HBV infection was observed among tribal populations in India, a fact that cannot be overlooked. This evidence base can serve as an advocacy tool to support increased funding, programmatic attention, and surveillance infrastructure for tribal health, contributing to India’s efforts to eliminate hepatitis. Proper diagnosis and estimation of the prevalence of this disease are essential to planning future vaccination strategies and managing HBV among tribal communities.
