# Supplementary material for: Hepatitis B Virus Infection Among Tribal Populations in India: A Systematic Review and Meta-Analysis
Source: Public Health Rev. 2026 Mar 2;46:1607620. doi: 10.3389/phrs.2025.1607620 (PMC12989728; doi:10.3389/phrs.2025.1607620)
Supplement: Supplementary file 1 [file Supplementaryfile1.docx]

**Supplementary Table S1: PRISMA reporting guidelines**

| **Section and Topic** | **Item #** | **Checklist item** | **Location where item is reported**  **(Page #)** |
| --- | --- | --- | --- |
| **TITLE** | | |  |
| Title | 1 | Identify the report as a systematic review. | Title |
| **ABSTRACT** | | |  |
| Abstract | 2 | See the PRISMA 2020 for Abstracts checklist. | Abstract |
| **INTRODUCTION** | | |  |
| Rationale | 3 | Describe the rationale for the review in the context of existing knowledge. | Introduction |
| Objectives | 4 | Provide an explicit statement of the objective(s) or question(s) the review addresses. | Introduction |
| **METHODS** | | |  |
| Eligibility criteria | 5 | Specify the inclusion and exclusion criteria for the review and how studies were grouped for the syntheses. | Eligibility of Studies |
| Information sources | 6 | Specify all databases, registers, websites, organisations, reference lists and other sources searched or consulted to identify studies. Specify the date when each source was last searched or consulted. | Information Sources |
| Search strategy | 7 | Present the full search strategies for all databases, registers and websites, including any filters and limits used. | Supplementary Table S2 |
| Selection process | 8 | Specify the methods used to decide whether a study met the inclusion criteria of the review, including how many reviewers screened each record and each report retrieved, whether they worked independently, and if applicable, details of automation tools used in the process. | Study Selection and Data Management |
| Data collection process | 9 | Specify the methods used to collect data from reports, including how many reviewers collected data from each report, whether they worked independently, any processes for obtaining or confirming data from study investigators, and if applicable, details of automation tools used in the process. | Study Selection and Data Management |
| Data items | 10a | List and define all outcomes for which data were sought. Specify whether all results that were compatible with each outcome domain in each study were sought (e.g. for all measures, time points, analyses), and if not, the methods used to decide which results to collect. | Study Selection and Data Management |
|  | 10b | List and define all other variables for which data were sought (e.g. participant and intervention characteristics, funding sources). Describe any assumptions made about any missing or unclear information. | Study Selection and Data Management |
| Study risk of bias assessment | 11 | Specify the methods used to assess risk of bias in the included studies, including details of the tool(s) used, how many reviewers assessed each study and whether they worked independently, and if applicable, details of automation tools used in the process. | Risk of Bias Assessment |
| Effect measures | 12 | Specify for each outcome the effect measure(s) (e.g. risk ratio, mean difference) used in the synthesis or presentation of results. | Data Synthesis |
| Synthesis methods | 13a | Describe the processes used to decide which studies were eligible for each synthesis (e.g. tabulating the study intervention characteristics and comparing against the planned groups for each synthesis (item #5)). | Data Synthesis |
|  | 13b | Describe any methods required to prepare the data for presentation or synthesis, such as handling of missing summary statistics, or data conversions. | Data Synthesis |
|  | 13c | Describe any methods used to tabulate or visually display results of individual studies and syntheses. | Data Synthesis |
|  | 13d | Describe any methods used to synthesize results and provide a rationale for the choice(s). If meta-analysis was performed, describe the model(s), method(s) to identify the presence and extent of statistical heterogeneity, and software package(s) used. | Data Synthesis |
|  | 13e | Describe any methods used to explore possible causes of heterogeneity among study results (e.g. subgroup analysis, meta-regression). | Data Synthesis |
|  | 13f | Describe any sensitivity analyses conducted to assess robustness of the synthesized results. | Data Synthesis |
| Reporting bias assessment | 14 | Describe any methods used to assess risk of bias due to missing results in a synthesis (arising from reporting biases). | NA |
| Certainty assessment | 15 | Describe any methods used to assess certainty (or confidence) in the body of evidence for an outcome. | NA |
| **RESULTS** | | |  |
| Study selection | 16a | Describe the results of the search and selection process, from the number of records identified in the search to the number of studies included in the review, ideally using a flow diagram. | Search Results |
|  | 16b | Cite studies that might appear to meet the inclusion criteria, but which were excluded, and explain why they were excluded. | Search Results |
| Study characteristics | 17 | Cite each included study and present its characteristics. | General characteristics of the included studies |
| Risk of bias in studies | 18 | Present assessments of risk of bias for each included study. | Table 1 |
| Results of individual studies | 19 | For all outcomes, present, for each study: (a) summary statistics for each group (where appropriate) and (b) an effect estimate and its precision (e.g. confidence/credible interval), ideally using structured tables or plots. | Prevalence of Hepatitis B |
| Results of syntheses | 20a | For each synthesis, briefly summarise the characteristics and risk of bias among contributing studies. | NA |
|  | 20b | Present results of all statistical syntheses conducted. If meta-analysis was done, present for each the summary estimate and its precision (e.g. confidence/credible interval) and measures of statistical heterogeneity. If comparing groups, describe the direction of the effect. | Prevalence of Hepatitis B |
|  | 20c | Present results of all investigations of possible causes of heterogeneity among study results. | NA |
|  | 20d | Present results of all sensitivity analyses conducted to assess the robustness of the synthesized results. | Prevalence of Hepatitis B |
| Reporting biases | 21 | Present assessments of risk of bias due to missing results (arising from reporting biases) for each synthesis assessed. | NA |
| Certainty of evidence | 22 | Present assessments of certainty (or confidence) in the body of evidence for each outcome assessed. | NA |
| **DISCUSSION** | | |  |
| Discussion | 23a | Provide a general interpretation of the results in the context of other evidence. | Key Findings |
|  | 23b | Discuss any limitations of the evidence included in the review. | Strengths and Limitations |
|  | 23c | Discuss any limitations of the review processes used. | Strengths and Limitations |
|  | 23d | Discuss implications of the results for practice, policy, and future research. | Implications for Policy and Practice |
| **OTHER INFORMATION** | | |  |
| Registration and protocol | 24a | Provide registration information for the review, including register name and registration number, or state that the review was not registered. | Methods: Standards and Protocols |
|  | 24b | Indicate where the review protocol can be accessed, or state that a protocol was not prepared. | Methods: Standards and Protocols |
|  | 24c | Describe and explain any amendments to information provided at registration or in the protocol. | NA |
| Support | 25 | Describe sources of financial or non-financial support for the review, and the role of the funders or sponsors in the review. | Funding |
| Competing interests | 26 | Declare any competing interests of review authors. | Conflict of Interest |
| Availability of data, code and other materials | 27 | Report which of the following are publicly available and where they can be found: template data collection forms; data extracted from included studies; data used for all analyses; analytic code; any other materials used in the review. | NA |

*From:* Page MJ, McKenzie JE, Bossuyt PM, Boutron I, Hoffmann TC, Mulrow CD, et al. The PRISMA 2020 statement: an updated guideline for reporting systematic reviews. BMJ 2021;372:n71. doi: 10.1136/bmj.n71

For more information, visit:<http://www.prisma-statement.org/>

**Supplementary Table S2:** **Search strategy used for various databases**

| **Database** | **Search strategy** |
| --- | --- |
| PubMed | "Prevalence"[MeSH Terms] OR "prevalence*"[Title/Abstract] OR "period prevalence*"[Title/Abstract] OR "point prevalence*"[Title/Abstract] AND "Hepatitis B"[MeSH Terms] OR "Hepatitis B"[Title/Abstract] OR "hepatitis b virus infection*"[Title/Abstract] OR "hepatitis b viral infection*"[Title/Abstract] OR "Hepatitis B Virus"[Title/Abstract] AND "Indigenous Peoples” [MeSH Terms] OR "indigenous people*"[Title/Abstract] OR "first nation people*"[Title/Abstract] OR "native people *” [Title/Abstract] OR "native born” [Title/Abstract] OR “native born” [Title/Abstract] OR "native born"[Title/Abstract] OR "indigenous population*"[Title/Abstract] OR "trib*"[Title/Abstract] |
| Embase | Prevalence/exp OR prevalence*:ti,ab OR 'period prevalence*':ti,ab OR 'point prevalence*':ti,ab AND 'Hepatitis B'/exp OR 'Hepatitis B':ti,ab OR 'hepatitis b virus infection*':ti,ab OR 'hepatitis b viral infection*':ti,ab OR 'Hepatitis B Virus':ti,ab AND 'Indigenous Peoples'/exp OR 'indigenous people*':ti,ab OR 'first nation people*':ti,ab OR 'native people *':ti,ab OR 'native born':ti,ab OR 'native born':ti,ab OR 'native born':ti,ab OR 'indigenous population*':ti,ab OR trib*:ti,ab |
| CINAHL | (MH Prevalence+) OR (TI prevalence* OR AB prevalence*) OR (TI "period prevalence*" OR AB "period prevalence*") OR (TI "point prevalence*" OR AB "point prevalence*") AND (MH "Hepatitis B+") OR (TI "Hepatitis B" OR AB "Hepatitis B") OR (TI "hepatitis b virus infection*" OR AB "hepatitis b virus infection*") OR (TI "hepatitis b viral infection*" OR AB "hepatitis b viral infection*") OR (TI "Hepatitis B Virus" OR AB "Hepatitis B Virus") AND (MH "Indigenous Peoples+") OR (TI "indigenous people*" OR AB "indigenous people*") OR (TI "first nation people*" OR AB "first nation people*") OR (TI "native people *" OR AB "native people *") OR (TI "native born" OR AB "native born") OR (TI "native born" OR AB "native born") OR (TI "native born" OR AB "native born") OR (TI "indigenous population*" OR AB "indigenous population*") OR (TI trib* OR AB trib*) |

**Supplementary Table S3:** **Detailed questions of AXIS tool used for risk of bias assessment**

| Introduction | |
| --- | --- |
| Q1 | Were the aims/objectives of the study clear? |
| Methods | |
| Q2 | Was the study design appropriate for the stated aim(s)? |
| Q3 | Was the sample size justified? |
| Q4 | Was the target/reference population clearly defined? (Is it clear who the research was about?) |
| Q5 | Was the sample frame taken from an appropriate population base so that it closely represented the target/reference population under investigation? |
| Q6 | Was the selection process likely to select subjects/participants that were representative of the target/reference population under investigation? |
| Q7 | Were measures undertaken to address and categorise non-responders? |
| Q8 | Were the risk factor and outcome variables measured appropriate to the aims of the study? |
| Q9 | Were the risk factor and outcome variables measured correctly using instruments/ measurements that had been trialled, piloted or published previously? |
| Q10 | Is it clear what was used to determined statistical significance and/or precision estimates? (eg, p values, CIs) |
| Q11 | Were the methods (including statistical methods) sufficiently described to enable them to be repeated? |
| Results | |
| Q12 | Were the basic data adequately described? |
| Q13 | Does the response rate raise concerns about non-response bias? |
| Q14 | If appropriate, was information about non-responders described? |
| Q15 | Were the results internally consistent? |
| Q16 | Were the results for the analyses described in the methods, presented? |
| Discussion | |
| Q17 | Were the authors’ discussions and conclusions justified by the results? |
| Q18 | Were the limitations of the study discussed? |
| Other | |
| Q19 | Were there any funding sources or conflicts of interest that may affect the authors’ interpretation of the results? |
| Q20 | Was ethical approval or consent of participants attained? |

**Supplementary Table 4: Diagnostic/Testing methods used to estimate the sero-prevalence of Hepatitis B infection**

| **Author Name and Year** | **Testing Method** |
| --- | --- |
| Anvikar AR et al., 2008 | ELISA |
| Baliarsingh B et al., 2017 | ELISA |
| Barall D et al., 2018 | ELISA |
| Bhattacharya H et al., 2014 | ELISA, PCR |
| Bhattacharya H et al., 2014 | ELISA, PCR |
| Bhattacharya H et al., 2015 | ELISA |
| Bhaumik P et al., 2014 | ELISA |
| Biswas D et al., 2007 | ELISA |
| Borkakoty BJ et al., 2008 | ELISA, PCR |
| Dinesh Ramalakshmi et al., 2017 | ELISA |
| Dwibedi B et al., 2014 | ELISA, PCR |
| Ghosh S et al., 2010 | ELISA |
| Gnanasekaran A et al., 2013 | ELISA |
| Habeeb MA et al., 2003 | EIA, PCR |
| Haldipur BP et al., 2014 | ELISA |
| Manjiyil IR et al., 2021 | ELISA |
| Murhekar MV et al., 2000 | ELISA |
| Murhekar MV et al., 2002 | ELISA |
| Murhekar MV et al., 2003 | ELISA |
| Murhekar MV et al., 2004 | ELISA |
| Reddy P.H. et al., 1995 | ELISA |
| Sharma RK et al., 2019 | ELISA |
| Shyamala R et al., 2016 | ELIFA |

*Enzyme-linked immunosorbent assay: ELISA; Enzyme Linked Immuno-Filtration Assay: ELIFA; and Enzyme immuno-assay: EIA

| 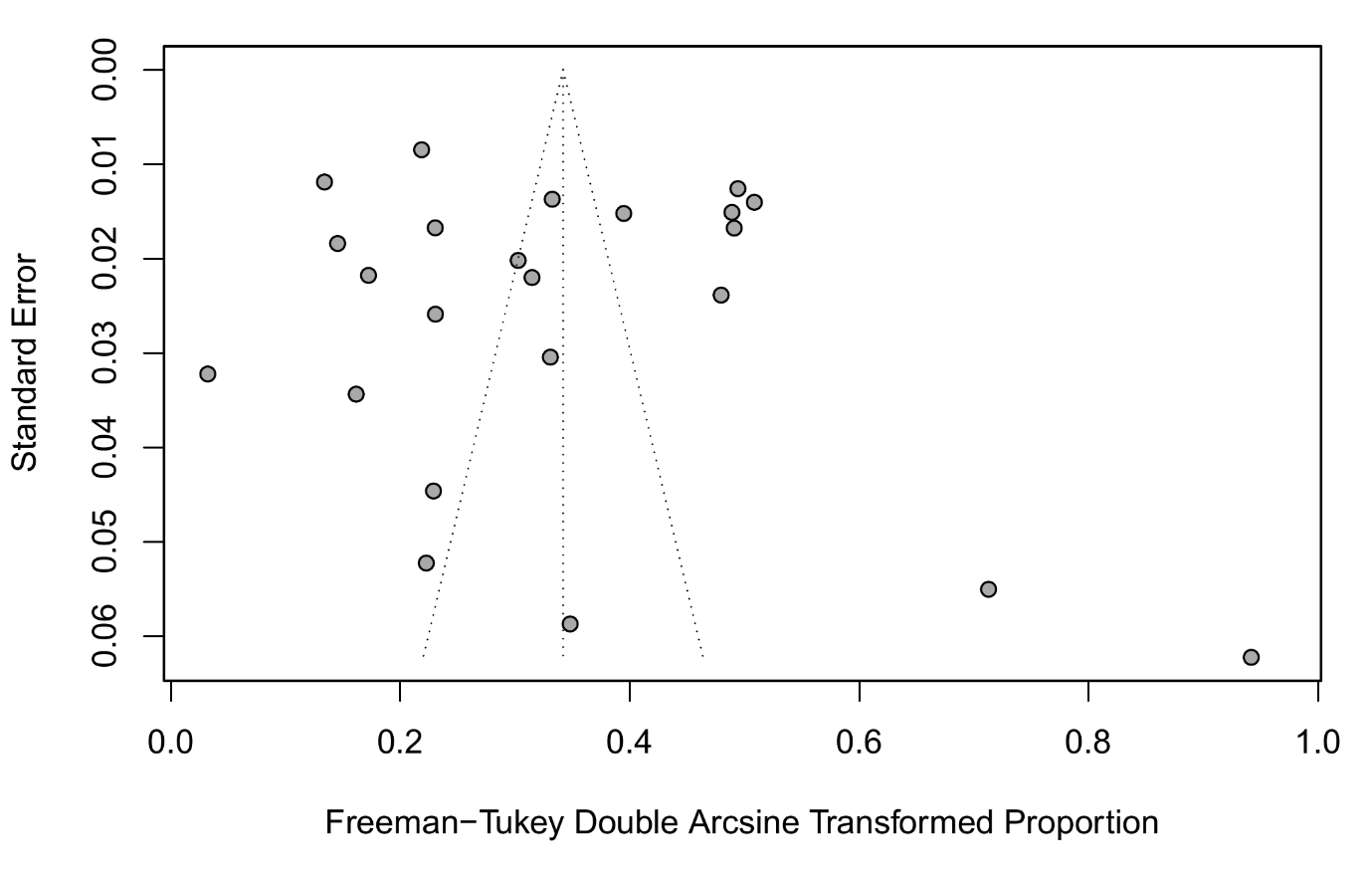 |
| --- |
| Supplementary Figure 1: Funnel plot  Note: Linear regression test of funnel plot asymmetry (t = 0.58, df = 21, Egger’s p-value = 0.5657) |

| 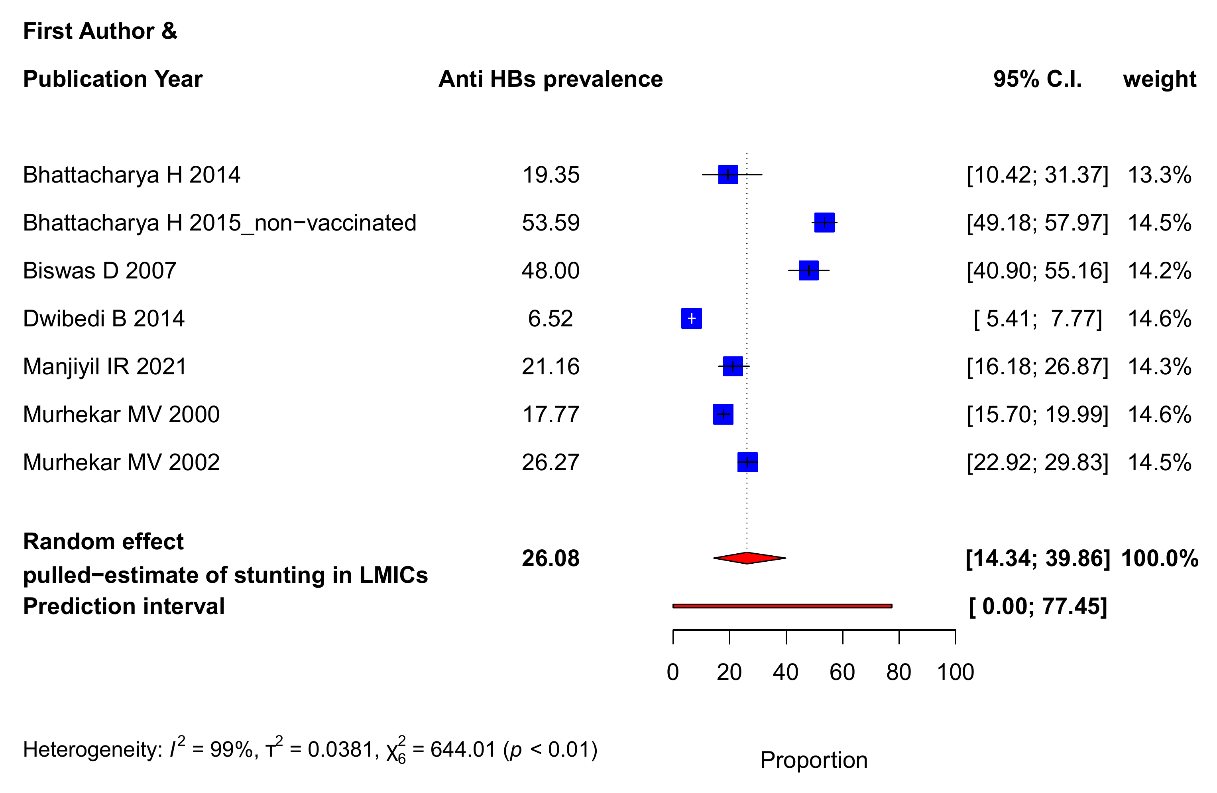 |
| --- |
| Supplementary Figure 2: Forest plot for pooled prevalence of anti-HBs. |
| 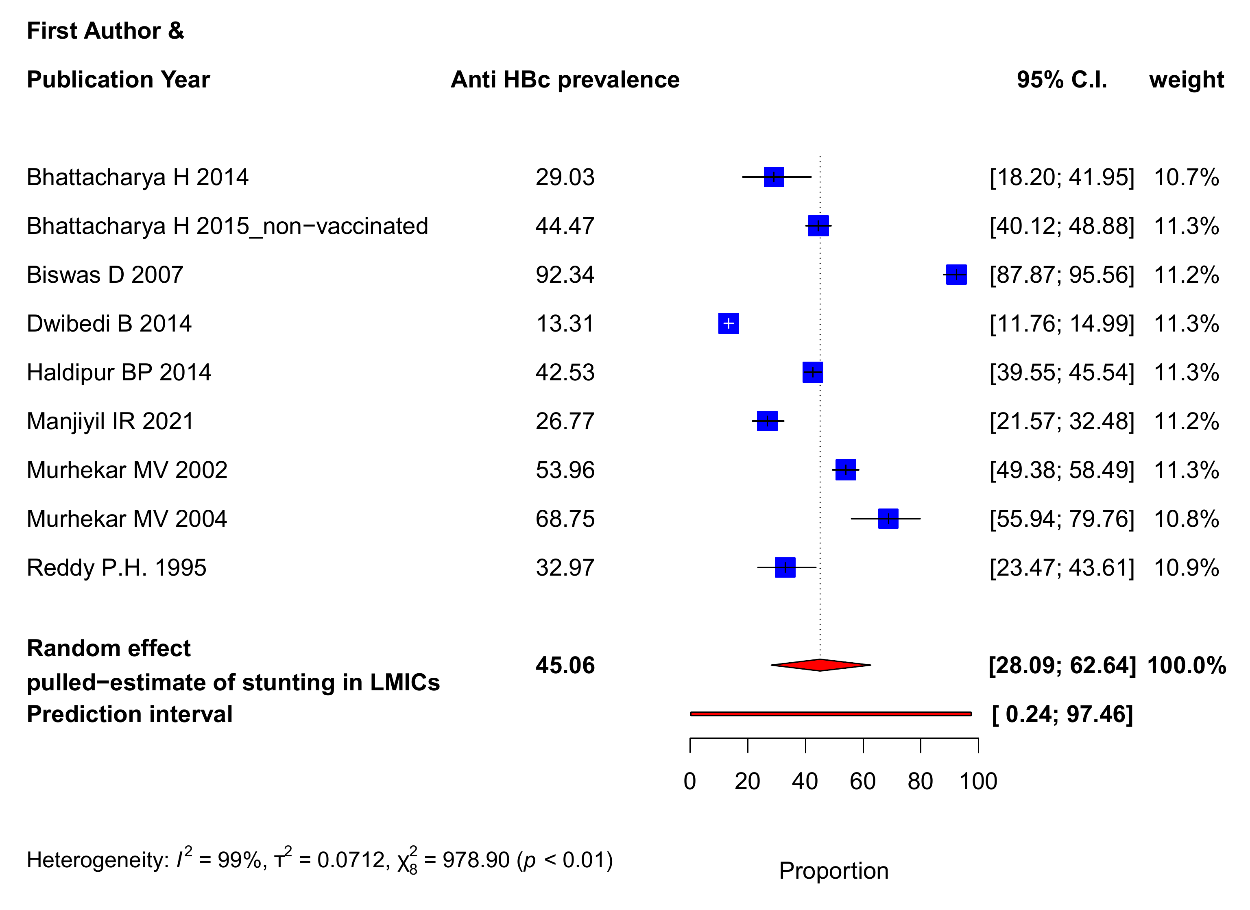 |
| Supplementary Figure 3: Forest plot for pooled prevalence of anti-HBc. |

**Supplementary Table 5: Risk of bias assessment in included studies**

| **Studies** | **Introduction** | **Methods** | | | | | | | | | | **Results** | | | | | **Discussion** | | **Other** | | **Decision** | |
| --- | --- | --- | --- | --- | --- | --- | --- | --- | --- | --- | --- | --- | --- | --- | --- | --- | --- | --- | --- | --- | --- | --- |
|  | Q1 | Q2 | Q3 | Q4 | Q5 | Q6 | Q7 | Q8 | Q9 | Q10 | Q11 | Q12 | Q13 | Q14 | Q15 | Q16 | Q17 | Q18 | Q19 | Q20 | Yes n (%) | Risk of Bias |
| Anvikar AR et al., 2008^12^ | Y | Y | Y | Y | Y | Y | N | Y | Y | Y | Y | Y | N | N | Y | Y | Y | N | N | Y | 15 (75) | Medium |
| Baliarsingh B et al., 2017^13^ | Y | Y | DK | Y | Y | Y | N | DK | DK | DK | N | Y | N | N | DK | N | Y | N | N | DK | 7 (35) | High |
| Barall D et al., 2018^14^ | Y | Y | Y | Y | Y | Y | N | Y | Y | Y | Y | Y | N | N | Y | Y | Y | N | N | Y | 15 (75) | Medium |
| Bhattacharya H et al., 2014^15^ | Y | Y | N | Y | Y | Y | N | Y | Y | Y | Y | Y | N | N | N | Y | Y | N | N | Y | 13 (65) | Medium |
| Bhattacharya H et al., 2014^16^ | Y | Y | Y | Y | Y | Y | N | Y | Y | Y | Y | Y | N | N | Y | Y | Y | Y | N | Y | 16 (80) | Medium |
| Bhattacharya H et al., 2015^17^ | Y | Y | Y | Y | Y | Y | N | Y | Y | Y | Y | Y | N | N | Y | Y | Y | N | N | Y | 15 (75) | Medium |
| Bhaumik P et al., 2014^18^ | Y | Y | Y | Y | Y | Y | N | Y | Y | Y | Y | Y | N | N | Y | Y | Y | Y | N | Y | 16 (80) | Medium |
| Biswas D et al., 2007^19^ | Y | Y | Y | Y | Y | Y | N | Y | Y | Y | Y | Y | N | N | Y | Y | Y | N | N | Y | 15 (75) | Medium |
| Borkakoty BJ et al., 2008^20^ | Y | Y | N | Y | Y | Y | N | Y | Y | N | N | N | N | N | N | Y | Y | N | N | Y | 10 (50) | High |
| Dinesh R et al., 2017^21^ | Y | Y | N | Y | Y | Y | N | Y | Y | N | N | Y | N | N | N | Y | Y | N | N | Y | 11 (55) | Medium |
| Dwibedi B et al., 2014^22^ | Y | Y | N | Y | Y | Y | N | Y | Y | Y | Y | Y | N | N | Y | Y | Y | N | N | Y | 14 (70) | Medium |
| Ghosh S et al., 2010^23^ | Y | Y | Y | Y | Y | Y | N | Y | Y | N | Y | Y | N | N | N | Y | Y | N | N | Y | 13 (65) | Medium |
| Gnanasekaran A et al., 2013^24^ | Y | Y | N | Y | N | N | N | Y | Y | N | Y | Y | N | N | N | Y | Y | N | N | Y | 10 (50) | High |
| Habeeb MA et al., 2003^25^ | Y | Y | N | N | N | DK | DK | DK | Y | N | N | N | DK | N | DK | N | Y | N | N | DK | 4 (20) | High |
| Haldipur BP et al., 2014^26^ | Y | Y | N | Y | Y | Y | N | Y | Y | Y | Y | Y | N | N | Y | Y | Y | Y | N | Y | 15 (75) | Medium |
| Manjiyil IR et al., 2021^27^ | Y | Y | Y | Y | Y | Y | N | Y | Y | Y | Y | Y | N | N | Y | Y | Y | Y | N | Y | 16 (80) | Medium |
| Murhekar MV et al., 2000^28^ | Y | Y | Y | Y | Y | Y | N | Y | Y | Y | Y | Y | N | N | Y | Y | Y | Y | N | Y | 16 (80) | Medium |
| Murhekar MV et al., 2002^29^ | Y | Y | Y | Y | Y | Y | N | Y | Y | Y | Y | Y | N | N | Y | Y | Y | Y | N | Y | 16 (80) | Medium |
| Murhekar MV et al., 2003^30^ | Y | Y | N | Y | Y | Y | N | Y | Y | Y | Y | Y | N | N | N | Y | Y | N | N | Y | 13 (65) | Medium |
| Murhekar MV et al., 2004^31^ | Y | Y | Y | Y | Y | Y | N | Y | Y | N | N | Y | N | N | Y | Y | Y | N | N | Y | 13 (65) | Medium |
| Reddy P.H. et al., 1995^32^ | Y | Y | N | Y | Y | Y | N | Y | Y | Y | Y | Y | N | N | N | Y | Y | Y | N | Y | 14 (70) | Medium |
| Sharma RK et al., 2019^33^ | Y | Y | Y | Y | Y | Y | N | Y | Y | Y | Y | Y | N | N | N | Y | Y | Y | N | Y | 15 (75) | Medium |
| Shyamala R et al., 2016^34^ | Y | Y | Y | Y | Y | Y | N | Y | Y | N | Y | Y | N | N | Y | DK | Y | Y | N | Y | 14 (70) | Medium |
| *The detailed description of each of the question (Q1-Q20) is provided in in the supplementary file ; *DK: Don’t Know | | | | | | | | | | | | | | | | | | | | |  | |
